# Mer overexpression in *Methanosarcina acetivorans* affects growth and methanogenesis during substrate adaptation

**DOI:** 10.1128/aem.00675-25

**Published:** 2025-04-25

**Authors:** Darla Brennan, Dillon Lieber, Mary Walter, Morgan Price, Nicole R. Buan

**Affiliations:** 1 Department of Biochemistry, University of Nebraska-Lincoln 315569, Lincoln, Nebraska, USA; Michigan State University, East Lansing, Michigan, USA

**Keywords:** methane, archaea, *Methanosarcina*, methanogenesis, Wood-Ljungdahl, one-carbon

## Abstract

Evidence suggests that multienzyme complexes are involved in biological methane production (methanogenesis), although the composition of the Wolfe Cycle methanogenesis complexes may vary between diverse methanoarchaeal taxa. Methylenetetrahydromethanopterin reductase (Mer) is the first committed step in C1 oxidation to CO_2_ during methylotrophic methanogenesis. However, Mer is downregulated when cells use acetate as a substrate. We hypothesized that Mer overexpression during methylotrophic methanogenesis would be beneficial, while overexpression during acetoclastic methanogenesis would be detrimental for energy conservation. To test this hypothesis, we overexpressed Mer and characterized strain physiology on methanol, acetate, and when switching substrates. We found that Mer overexpression results in faster growth on methanol, with less C fixation into biomass, and no effect on methanogenesis. Growth on acetate was not affected by Mer overexpression, but switching between substrates was affected. The native Mer overexpressing strain was slower to adjust from methanol to acetate and vice-versa. These data suggest that tight regulation of Mer expression is necessary to regulate C flux through methylotrophic versus acetoclastic methanogenesis pathways in *Methanosarcina*.

**IMPORTANCE:**

Methanoarchaea thrive near the “thermodynamic limit of life” and have likely evolved efficient mechanisms to control flux of substrates to conserve energy. Methylenetetrahydromethanopterin reductase (Mer) is a highly conserved, key enzyme in the Wood-Ljungdahl and Wolfe Cycle methanogenesis pathways. Our study sheds light on how Mer enzyme stoichiometry affects methanogenesis and suggests avenues for engineering the organism to promote renewable fuel or bioproduct synthesis.

## INTRODUCTION

Methanogens are strictly anaerobic archaea that are able to obtain the energy they need for growth through methanogenesis, in which carbon substrates are reduced to methane (1, 2). These organisms are key players in the global carbon cycle in which they contribute ~1 Gt of CH_4_ annually in anaerobic environments (3). Due to their role in global methane production, methanogens have been used in anaerobic digesters and wastewater facilities for the production of biogas and provide intriguing potential in metabolic engineering toward the production of other renewable bioproducts and fuels (4, 5). Methanogens are able to use a range of carbon sources such as methanol, acetate, formate, CO, CO_2_, methylamines, methyl-sulfides for methanogenesis (6), and recent investigations suggest methanoarchaea can also use coal or lignin monomers and other alkanes (7–10). The directionality of electron flux through methanogenesis pathway enzymes depends on the carbon source that is used (11). For example, during hydrogenotrophic methanogenesis, CO_2_ is reduced to methane using hydrogen gas as a reductant, whereas during methylotrophic growth, methanol is oxidized to CO_2_, which results in a reduced ferredoxin and reduced F_420_ and provides electrons necessary for energy conservation and synthesis of acetyl-CoA (12–14). During acetoclastic growth, the cleavage of acetyl-CoA provides electrons that result in the reduction of ferredoxin, which can then be oxidized to conserve energy (13).

Energy conservation occurs in methanogens by coupling the channeling of electrons from reduced electron carriers to the CoM-S-S-CoB terminal electron acceptor while simultaneously generating a transmembrane ion gradient (13). The CoM-S-S-CoB heterodisulfide is then reduced by either a cytosolic (HdrABC) or membrane-bound (HdrED) heterodisulfide reductase to generate a cyclic pathway known as the Wolfe Cycle (15–17). The channeling of such electrons and substrates between methanogenesis steps is believed to occur through the use of multienzyme complexes, which would prevent the diffusion of intermediates, increasing the rate of the reactions and decreasing the chemical entropy (18–20). Previous studies indicate that in *Methanosarcina acetivorans,* the energy-conserving heterodisulfide reductase (HdrED) forms a complex with other key methanogenesis enzymes, acetyl-CoA decarbonylase/synthase (ACDS) and methylenetetrahydromethanopterin (Mer). This evidence suggests that the methanogen *M. acetivorans* may be able to use the Hdr:ACDS:Mer multienzyme complex as “redox router” to coordinate carbon fixation and energy conservation (20).

The enzyme ACDS is responsible for either acetyl-CoA synthesis under methylotrophic conditions or the cleavage of acetyl-CoA to produce CO_2_ under acetoclastic conditions, and Mer is the essential first step of the oxidative branch of the methylotrophic methanogenesis pathway (21–23). The ACDS complex is also often designated as the Cdh/ACS complex due to its separate carbon monoxide dehydrogenase (Cdh) and acetyl-CoA synthase (ACS) functioning subunits, as well as a high degree of conservation between methanoarchaea and acetogenic bacterial species (24–26). The HdrED catalytic subunits, HdrD, which was found to be interacting with ACDS in the Hdr:ACDS:Mer complex (20), also appears to resemble a fusion protein of the HdrBC catalytic subunits (26, 27). So while interactions between HdrED and ACDS have been identified, there is also a possibility that HdrABC may bind with ACDS, though evidence of this interaction has not yet been reported.

While multienzyme complexes are essential for energy conservation by channeling substrates and electrons, the formation of such complexes likely varies between methanogen species as the expressed proteins often differ between methanogen taxa (18, 19, 28). This biochemical flexibility suggests that there is an opportunity to engineer methanogenic pathways to improve the growth kinetics of these organisms. As the enzymes in the Hdr:ACDS:Mer complex are involved in coordinating C fixation vs methanogenesis, we wonder if manipulating the stoichiometries of these enzymes and their components could have an effect on the rates of methanogenesis or biomass synthesis. With acetoclastic growth, Mer and the oxidative branch are transcribed at low levels (29) but have been shown to remain essential for growth under these conditions (30). At the same time, Acs/Cdh expression is greatly enhanced. Previous work explored Hdr (31) and Cdh (32) essentiality and growth effects. We expand on these studies to explore the effects of Mer overexpression on *M. acetivorans* physiology.

Currently, efforts to engineer methanogens to synthesize high-value bioproducts or biofuels are hindered by an incomplete knowledge of how carbon and electron flows are regulated by the cell. We hypothesized that increased expression of Mer, an enzyme involved in an ancient multienzyme complex, would affect growth, methanogenesis rates, and biomass efficiency, or allow cells to adapt more readily to changing substrate conditions. We report that overexpression of Mer caused a decrease of carbon fixation into biomass and faster growth on methanol, though no effects on growth or methanogenesis were observed during acetoclastic growth. Mer overexpression does, however, cause significant changes to growth and methanogenesis rates when switching substrate conditions, particularly when switching from acetoclastic to methylotrophic conditions. This suggests that regulation of Mer expression in *Methanosarcina* is necessary in controlling carbon flux through the oxidation branch of methylotrophic versus acetoclastic methanogenesis pathways.

## MATERIALS AND METHODS

### Culturing conditions

*M. acetivorans* strains used in these experiments (Table 1) were grown under strictly anaerobic conditions in a custom B-type Coy anaerobic chamber (32; Coy Labs, Grass Lake, MI) with the internal environment maintained at 5% H_2_/20% CO_2_/75% N_2_ (Matheson Gas, Lincoln, NE, USA). The strains were inoculated into 10 mL of high-salt (HS) media (33) supplemented with either methanol (125 mM) or acetate (120 mM) as the carbon source, adapted to methanol then switched to acetate media, or adapted to acetate media and then switched to methanol (33, 34). For cells that were adapted to methanol or acetate before switching the carbon source, cells were adapted over a minimum of five cell passages (30 generations) before switching to HS media supplemented with the different C source. Cultures were grown in glass Balch tubes (Bellco Glass, Vineland, NJ, USA) to maintain anaerobic conditions once removed from the chamber. Once inoculated, cells were incubated at 37°C without shaking in a Thermo Scientific MaxQ 6000 Incubated/Refrigerated Stackable Shaker incubator (Thermo Fisher Scientific, Waltham, MA, USA). For cells grown on solid medium, HS medium with 1.4% agar added was used, and plates were incubated under premixed 20% CO_2_/79.9% N_2_/0.1% H_2_S gas (Matheson Gas) as previously described (35). All chemicals supplied by ThermoFisher (Waltham, MA) or Sigma-Aldrich (Burlington, MA).

**TABLE 1:** Strains, plasmids, and primers used in this study

Strain, plasmid, or primer	Description	Purpose	Source
*M. acetivorans* strains			
NB34	Δ *hpt::PmcrB-tetR*/ϕC31 *int/att*B	Parent	WWM82 (36)
NB42	Δ *hpt::PmcrB-tetR*/ϕC31 *int, att:*pNB662	*Pmcr hdrD2*^strep^ expression	WWM82 (36)
NB210	Δ *hpt::*ϕC31 *int, att::*pNB746	Constitutive MA3733 Mer native	This study
NB232	Δ *hpt::*ϕC31 *int, att:*pSK2	Control for testing dual-tagged ^strep-his^UidA^his-strep^ expression from pNB730	(35)
NB249	Δ *hpt::*ϕC31 *int, att:*pMW1	Constitutive MA3733 Mer-^his-strep^	This study
Plasmids			
pNB730	pUC *ori bla PmcrB pac(opt) ϕ*C31 *attB*	*Methanosarcina* spp. integration and expression vector	(35)
pNB746	pNB730: NdeI MA3733 BamHI	*M. acetivorans* expression of native MA3733 (*mer*)	This study
pMW1	pNB730: NdeI MA3733 -stop BamHI	*M. acetivorans* expression of C-tagged MA3733^his-strep^	This study
Primers			
oNB236	ATTAAGGAGGAAATTCATATGAAGTTCGGAATCGAATTTGTGCC	Amplifies MA3733 Mer for pNB730, NdeI fwd	This study
oNB237	CGAGGGCCCAAGCTTGGATCCTTACATCTTTGCAATGATCTCTTTGCC	Amplifies MA3733 Mer for pNB730, rev	This study
oNB238	CGAGGGCCCAAGCTTGGATCCttCATCTTTGCAATGATCTCTTTGCCTATAAGCTT	Amplifies MA3733 Mer for pNB730, -stop rev	This study
oNB380	aaaaaaaaaaaaCATATGtggtcccatcctcagtttgaaaaaAAGTTCGGAATCGAATTTGTGCC	Amplifies ^strep^MA3733 Mer, NdeI fwd	This study
oNB381	aaaaaaaaaaaaGGATCCTTAtttttcaaactgaggatgggaccaCATCTTTGCAATGATCTCTTTGCC	Amplifies MA3733^strep^ Mer, BamHI rev	This study
oNB52	GAAGCTTCCCCTTGACCAAT	Screening for integration of plasmids at ϕC31 *int attB/P hpt* locus	(36)
oNB53	TTGATTCGGATACCCTGAGC	Screening for integration of plasmids at ϕC31 *int attB/P hpt* locus	(36)
oNB54	GCAAAGAAAAGCCAGTATGGA	Screening for integration of plasmids at ϕC31 *int attB/P hpt* locus	(36)
oNB317	GATGAGTGGCAGGGCGGGGCGTAAT	Screening for integration of plasmids at ϕC31 *int attB/P hpt* locus	(35)
oN443	TGGAACATTTCCAGCTTTAAAAGCGAGCTTTGCACCTGCGAACTTGACC	Internal MA3733 for sequencing, rev	This study
oNB448	CGTCTGCAACTTCACCGGCA	Internal MA3733 for sequencing, rev	This study
oNB449	TGCAGGGTTCCTGGTATAGG	Internal MA3733 for sequencing, rev	This study
oNB450	CAATTTACATGGGTGCTCAG	Internal MA3733 for sequencing, fwd	This study

### Plasmids and strain construction

Genetic manipulation methods for *M. acetivorans* have been described previously (35). PCR primers were synthesized by Integrated DNA Technologies (IDT, Coralville, IA, USA), and PCR amplification of genes of interest was performed using the primers listed in Table 1 with Phusion Flash PCR Master Mix (Thermo Fisher Scientific, Waltham, MA, USA). The resultant PCR products were cloned into the pNB730 vector plasmid at NdeI and BamHI restriction sites using enzymes from Thermo Fisher Scientific (Waltham, MA, USA). Expression of the tagged Mer was achieved using oligos which fused the codon-optimized strep-tag II peptide to the 3′ end of the gene coding sequence (37). Following transformation in *Escherichia coli*, the resulting plasmids were transfected into *M. acetivorans* using 1,2-dioleoyl-3-trimethylammonium propane (DOTAP) liposomal transformation as previously described (38, 33, 39). All plasmids were screened by PCR using the primers listed in Table 1 and verified by sequencing (36, Eurofins, Louisville, KY, USA).

### PAGE and immunolotting

Samples were visualized on 15-µl well 4%–20% gradient SDS-PAGE gels, run at 100V for 1 h with 1× tris glycine SDS (TGS) running buffer in a Mini-PROTEAN Tetra Cell (Bio-Rad, Hercules, CA, USA). Proteins were transferred to an ImmunoBlot PVDF Membrane (BioRad, USA) at 100V for 1 h. Detection of proteins was then performed according to the SNAP I.D. 2.0 systems protocols (Sigma Aldrich [40]). Primary antibody was either custom rabbit anti-Mer (raised against peptide sequence CDLVLERHGIPVDAKKQIGD) from Life Technologies (Pittsburgh, PA) or Strep-Tag II Antibody HRP Conjugate (Novagen). Sheep Anti-Mouse IgG HRP-Linked Antibody (GE Healthsciences) was used as the secondary antibody, and Clarity Western ECL Substrate (BioRad, USA) was used to detect the HRP signal.

### Cell growth measurements

The optical density of the cultures was measured at 600 nm using a Spectronic D spectrophotometer (Thermo Fisher Scientific, Waltham, MA, USA) to determine the growth rate of the cell populations. Cultures were measured upon inoculation (0 h) until they reached a stable OD_600_ in stationary phase (unchanged OD_600_ for 24 h). Growth rates were calculated separately for each individual replicate culture, averaged, and two-tailed Student’s *t*-test was applied to assess statistical significance at the 95% confidence interval (*P* < 0.05).

### Biomass measurements

Cells were grown to stationary phase at 37°C until OD_600_ ~0.7 methanol-grown or OD_600_ ~0.11 for acetate-grown cells. The cells were then collected on dried and weighed Durapore 0.22-µm PVDF membranes (GVWP09050, MilliporeSigma) using vacuum filtration. Cells were then dried at 80°C for 3 days in a Precision Economy Oven (Precision Scientific, Chicago, IL). The dried filters were weighed and then returned to the oven to repeat 3 days of drying and weighing to ensure evaporation of all moisture.

### Methanogenesis assays

Each strain was grown to early stationary phase (OD_600_ ~0.3 for methanol grown or OD_600_ ~0.09 for acetate grown cells) in 10-mL cultures and brought into an anaerobic chamber on ice. Samples were centrifuged at 1,228 × *g* using an IEC Medilite Microcentrifuge (Thermo Fisher Scientific, Waltham, MA, USA). Cells were washed once with 10 mL of 3-(N-morpholino)propanesulfonic acid high-salt media (MHS) (41), resuspended in 4-mL MHS medium and kept cold on ice (42). Five autosampler vials of 500-µL cell suspensions were prepared per sample as replicates, as well as a medium-only control and a cells-only control (without a carbon source). For the sample replicates and medium-only control, 250 µL of 2xC MHS was added to each autosampler vial. For the medium-only control and cells-only control, 250 µ of MHS media was added to each vial. Then, 250 µL of cell suspension was added to the sample replicates and cells-only control vials. The autosampler vials were stoppered and crimped before removing from the chamber. The autosampler vials were then placed in an incubator at 37°C for 15 minutes to start the assay. Sample vials for each strain were removed from the incubator, and the CH_4_ in the headspace was quantified by gas chromatography using a flame ionization detector (GC-FID) as previously described (41).

### Calculation of metabolic efficiency

The metabolic efficiency *e* of the strains was determined by the relationship between the growth rate (*k*_g_) and rate of methanogenesis (*k*_CH4_) according to (43). The resulting values were normalized to the parent strain. To calculate metabolic efficiency, the following equation was used:


$$Percent\;e=\frac{k_{g}}{k_{CH4}}\;\times 100$$

## RESULTS

### Construction of Mer overexpression strains in *M. acetivorans*

**Fig 1:**
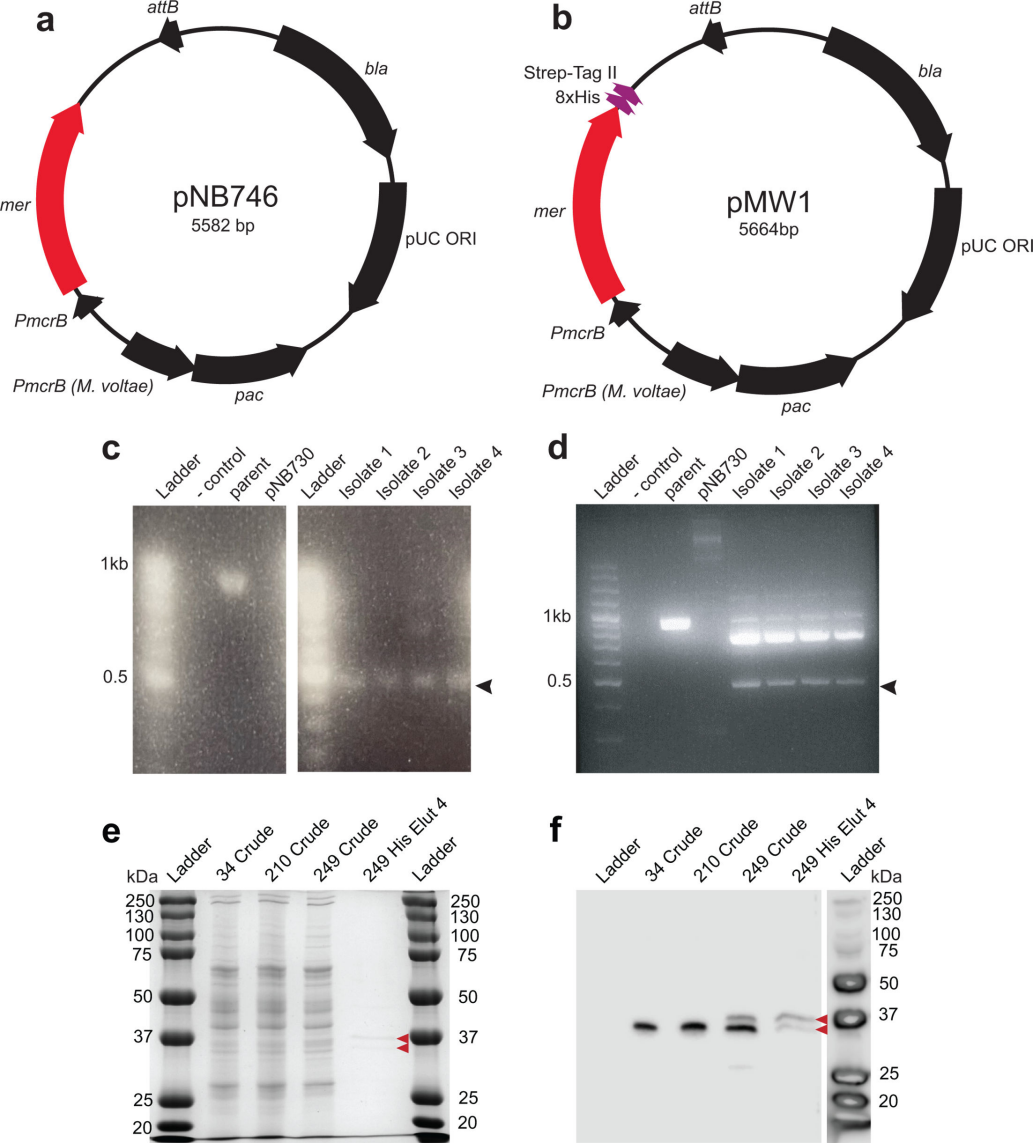
Construction of pNB746, pMW1, and strain validation. (a) Plasmid map of pNB746 for *att:mer^+^* strain construction and (b) plasmid map of pMW1 for obtaining the *att:mer^his-strep+^* strain. Following transformation into *M. acetivorans*, isolates for *att:mer^+^* (NB210 [c]) and *att:mer^his-strep+^* (NB249 [d]) strains were screened by diagnostic PCR. Black arrows show bands indicating expected sizes for plasmid integration. (e) SDS-PAGE of 3 µg of crude extracts from parent (NB34), *att:mer^+^* (NB210), *att:mer^+his-strep^* (NB249), and 1 µg of His-NTA purified Mer from NB249 analyzed on a 4% stacking/12.5% separating gel and stained with Coomassie Blue. (f) Immunoblot of the gel from panel a incubated with custom anti-MA3733 rabbit antibody and anti-rabbit HRP conjugated antibody for qualitative analysis of Mer overexpression (*att:mer^+^*). Red arrows indicate position of native and his-tagged Mer.

To determine the effects of Mer overexpression, plasmids pNB746 (Fig. 1a) and pMW1 (Fig. 1b) were constructed by cloning the amplified Mer gene (MA3733) from the *M. acetivorans* C2A chromosome into an overexpression vector, pNB730 (Table 1; 35). Following transfection, the resulting native overexpression plasmid (pNB746) and the C-terminal his-strep-tagged plasmid (pMW1) were integrated onto the *M. acetivorans* chromosome. Successful integration of the plasmids onto the chromosome was validated by PCR screen (Fig. 1c and d) as well as by SDS-PAGE and immunoblot for Mer protein (Fig. 1e and f). A strain which expresses dual-strep-his-tagged β-glucuronidase (*uidA*) reporter gene (NB232, Table 1) was used as a control for detecting expression of Mer^his-strep^ by immunoblot (Fig. S1). The expression levels of Mer in each of the strains were assessed by immunoblotting using a custom MA3733 antibody. Strain NB210 showed increased expression compared to the parent strain, indicating that Mer protein was successfully overexpressed (Fig. 1f; Fig. S1). Immunoblotting with purified Mer^his-strep^ protein also revealed native untagged Mer copurifies with overexpressed Mer^his-strep^, suggesting dynamic hetero-oligomers are formed *in vivo*.

### Overexpression of Mer affects growth rate and biomass productivity of substrate-adapted cells

**Fig 2:**
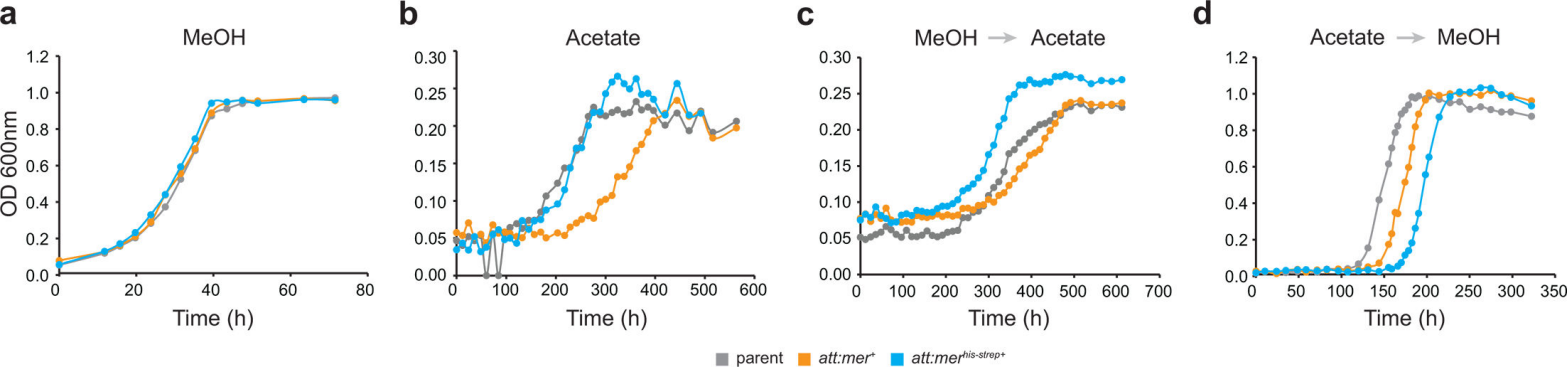
Growth curves of parent versus Mer overexpression strains. (a) Methanol-adapted growth curve. (b) Acetate-adapted growth curve. (c) Growth curves when methanol-adapted cells are inoculated into acetate. (d) Growth curves when acetate-adapted cells are inoculated into methanol. Each curve represents an average of 10 biological replicates. Error bars are omitted for clarity.

**Fig 3:**
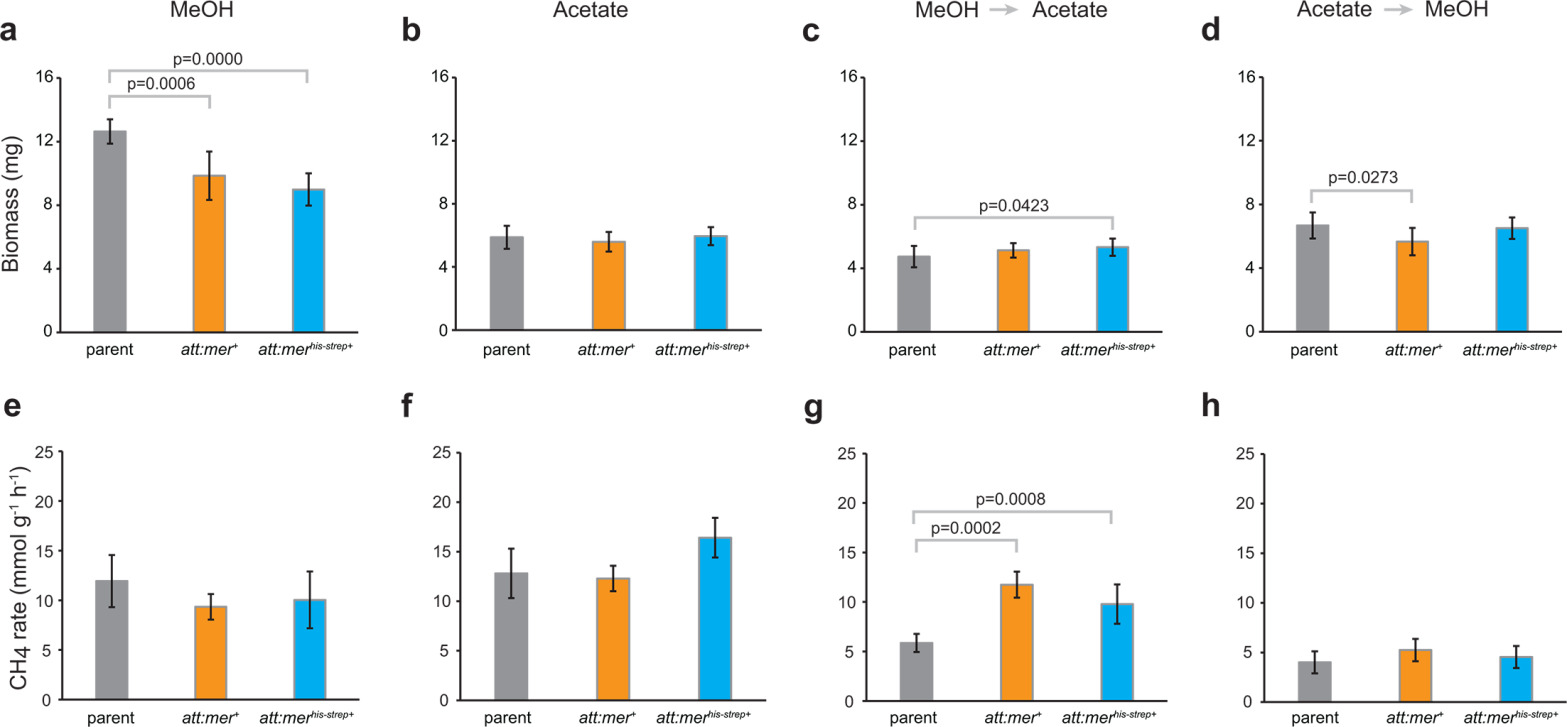
Effect of Mer overexpression on biomass production and rate of methanogenesis. (a–d) Biomass produced by parent and Mer overexpression strains when adapted to grow on methanol (a), on acetate (b), or when switched from methanol to acetate (c) or from acetate to methanol (d). Each measurement was obtained from 10 biological replicates. (e–h) Rate of methane produced by parent and Mer overexpression strains when adapted to grow on methanol (e), on acetate (f), or when switched from methanol to acetate (g) or from acetate to methanol (h). Each measurement was obtained from two biological and four to five technical replicates (*n* = 9). Error bars represent standard deviation.

**Fig 4:**
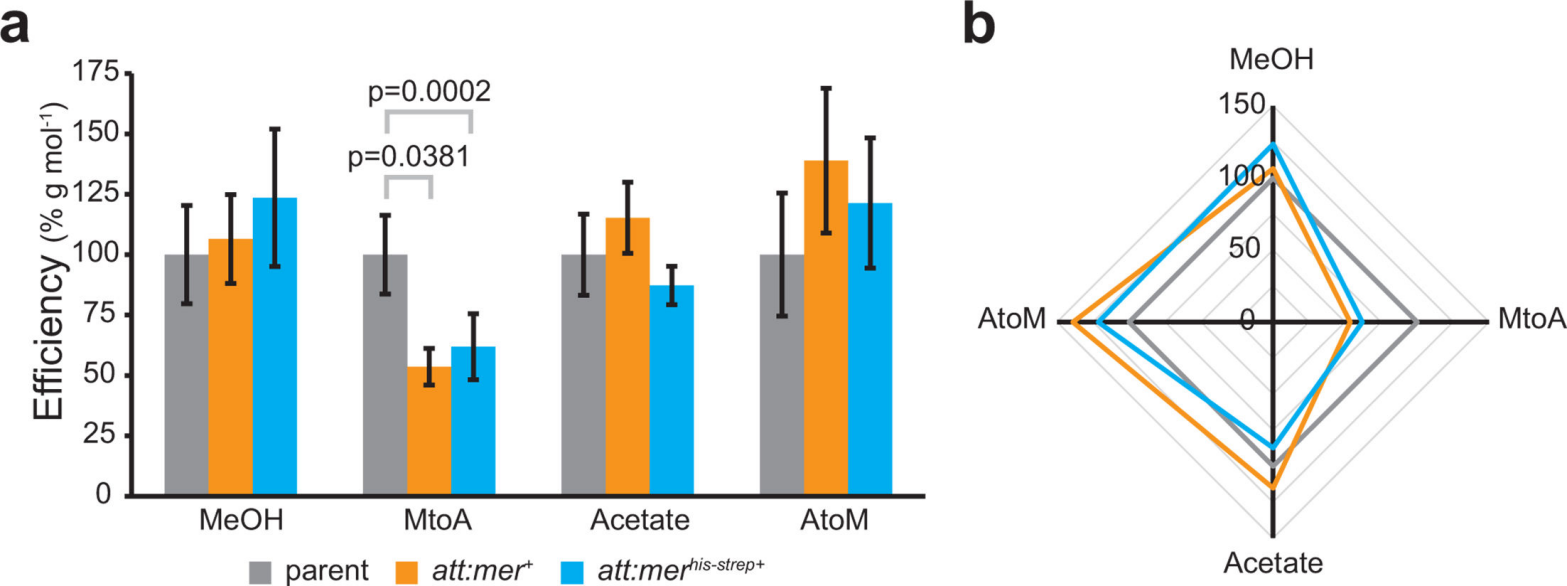
Metabolic efficiency of Mer overexpression strains versus parent strain. (a) Relative metabolic efficiency of parent and overexpression strains when adapted to methanol or acetate and when switched from methanol to acetate or acetate to methanol. (b) Two-dimensional hysteresis representation of relative metabolic efficiency indicating Mer overexpression primes metabolism for methylotrophic methanogenesis.

Cells were adapted to either methanol or acetate for 30 generations on each carbon source to allow for stabilization of the transcription profile when grown in the presence of that C source. When grown on methanol, NB210 (*att:mer^+^*) and NB249 (*att:mer^his-strep+^*) grew 13% and 8% faster (*P* = 0.002 and 0.010, respectively) compared to the parent strain (Fig. 2a; Table S1). While there were no significant changes in the rate of methane produced by mutant strains, there was a decrease in biomass production (22% and 29%, *P* = 0.001 and 0.000001, respectively) versus the parent strain under methylotrophic conditions (Fig. 3a; Tables S2 and S3). Overexpression of Mer (NB210) under acetoclastic conditions appeared to result in a slightly slower average growth rate (Fig. 2b); however, the decrease was not statistically significant (Table S1), likely due to small stochastic differences in the initial population size during inoculation. Mer overexpression also had no effect on biomass or methane production rates (Fig. 2b and 3b and f; Tables S2 and S3), consistent with this interpretation. No significant changes were observed to the overall metabolic efficiency of the strains when adapted to acetate or methanol (Fig. 4a and b; Table S4). However, there was a significant 27% decrease in biomass productivity in strains expressing tagged Mer (NB249) when growing on acetate (*P* = 0.032) (Fig. 4c and d; Table S4), and the cells were also shown to be significantly smaller than the parent strain (NB34) (Fig. S3 and S4).

### Overexpression of Mer causes significant changes when switching between substrates

To observe the effects of switching between carbon sources, each of the strains was first adapted to either methanol or acetate conditions by growing them in the presence of that carbon source for 30 generations. Once adapted, cells were then grown on the other carbon source for only five generations to observe the effects of switching substrates on Mer-dependent flux through the oxidative branch of the methanogenesis pathway. The strains that were first adapted to methanol and then switched to acetate showed drastic changes in growth and physiology. While NB210 (*att:mer^+^*) grew 75% slower (*P* = 0.00003) (Fig. 2c), slightly more biomass was produced, and the rate of methanogenesis was two times faster (Fig. 3c and g). The NB249 (*att:mer^his-strep+^*) strain produced 13% more biomass (*P* = 0.042) and had 36% faster methane production rates (*P* = 0.0008) than the parent strain (Fig. 3c and g). The metabolic efficiency of the Mer overexpression strains decreased (47% and 39%, *P* = 0.00005 and 0.0003) compared to the parent strain when switching from methanol to acetate (Fig. 4a and b). NB210 also showed a 48% decrease (*P* = 0.0047) in biomass productivity under these conditions (Fig. 4c and d). When switching from acetate to methanol, the native Mer overexpression strain (NB210) grew 50% slower (*P* = 0.025) and produced 16% less biomass (*P* = 0.022) than the parent strain (Fig. 2d and 3d). The Mer^his-strep^ strain (NB249) grew slightly slower, and both strains had slightly faster methanogenesis rates than the parent strain (Fig. 3d and h). There was also a slight increase in the metabolic efficiency as a result of Mer overexpression, although the increase was not statistically significant (Fig. 4a and b). However, NB210 showed a 36% decrease in biomass productivity (*P* = 0.0348) (Fig. 4c and d). Following growth under both switching conditions, NB210 and N249 cells were also shown to be slightly smaller than the parent strain (NB34) (Fig. S3 and S4; Table S5).

## DISCUSSION

**Fig 5:**
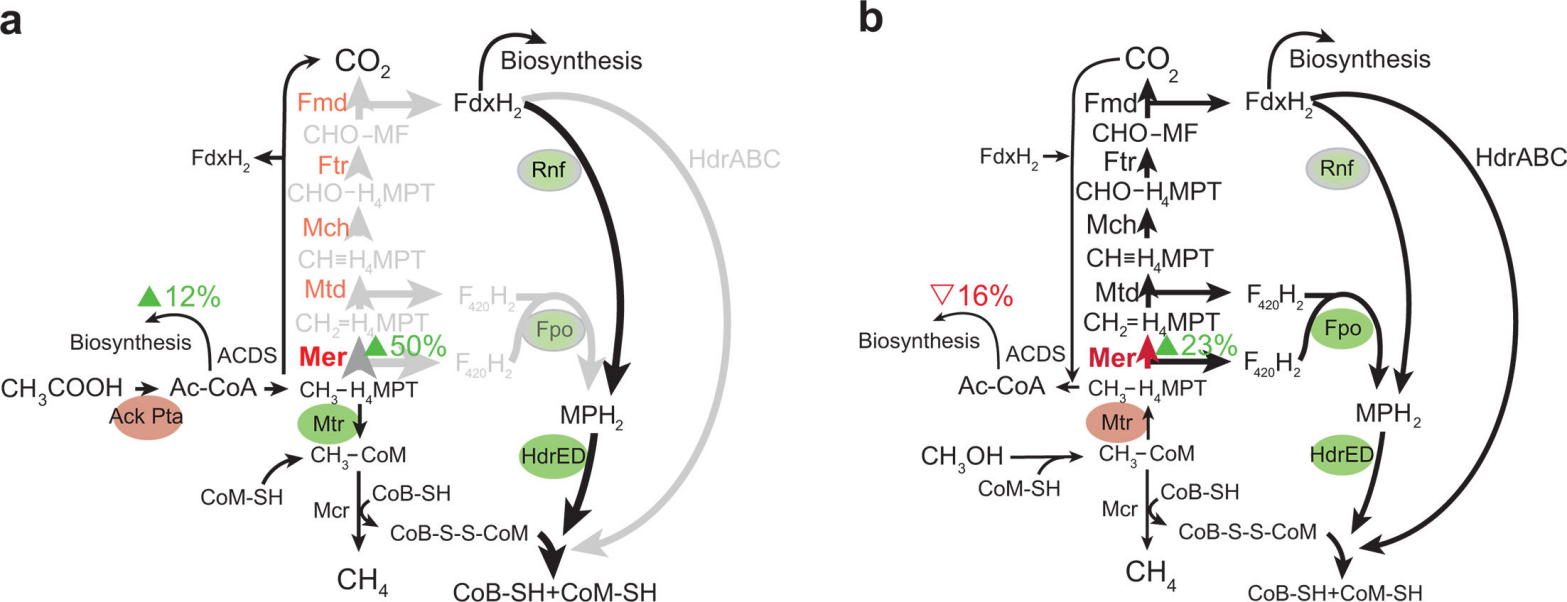
Effect of Mer overexpression when switching substrates. Inferred flux through methanogenesis pathways in the Mer overexpression strain when switching from methanol to acetate (a) and when switching from acetate to methanol (b). Arrow sizes are according to percent change in flux through pathway. Green ovals represent energy-conserving steps. Red ovals represent energy-consuming steps. Ac-CoA, acetyl-coenzyme A; ACDS, acetyl-CoA decarbonylase/synthase; Fmd, formyl-methanofuran dehydrogenase; Fpo, proton-pumping F_420_-methanophenazine oxidoreductase; Ftr, formyl-methanofuran:H_4_MPT formyl transferase; Hdr, heterodsulfide reductase; Mch, methenyl-H_4_MPT cyclohydrolase; Mcr, methyl-coenzyme M reductase; Mer, methylenetetrahydromethanopterin; Mtd, F_420_-dependent methylene-H_4_MPT dehydrogenase; Mtr, methyl-coenzyme M methyltransferase; Rnf, *Rhodobacter* nitrogen fixation.

Our observations show overexpression of Mer has a significant effect on metabolism in *M. acetivorans*. Increasing expression of Mer on methanol or acetate does not affect flux through the oxidative branch but affects it when switching from one substrate to the other (Fig. 5). On methanol, although the rates of growth and methane production were not affected by increased Mer expression, there was a decrease in biomass production and faster growth rates. This suggests that Mer overexpression diverts reducing equivalents to the oxidative branch at the expense of acetyl-CoA production via Cdh/ACS, which could be accomplished by increased flux through F_420_H_2_ and CH_2_ = MPT. The electrons, or indirectly the ATP produced as a result, could be balanced via non-energy-conserving pathways that do not contribute to biomass synthesis or the transmembrane ion gradients necessary for energy conservation, such as via HdrABC or an unknown futile cycle (involving electron carriers or ATP). Under methylotrophic and switching conditions, as a result of Mer overexpression, there is an increase in flux toward the oxidative branch rather than toward biomass production, leading to a decrease in biomass and cell size (Fig. S3 and S4). On acetate, biomass production, growth, and methane production rates were similar to the parent strain. As strains have adapted to the carbon source over 30 generations, it is possible that the cells have had sufficient time over several generations to adjust gene expression, leading to similar rates as observed in the parent strain.

When switching from methanol to acetate, increased expression of tagged Mer resulted in an increase in biomass and faster methanogenesis (Fig. 3c and g). This is presumably because the switch to growth on acetate typically results in decreased expression of Mer. In this case, Mer levels remain high and thus allow higher flux through oxidation of methyl-H_4_MPT, which leads to increased ATP energy conserved, higher biomass, and more flux through the oxidative branch of methanogenesis occurs, albeit with decreased efficiency (Fig. 4 and 5a). While the tagged overexpression strain (NB249, *att:mer^his-strep+^*) had a similar growth rate to the parent strain (NB34), the untagged overexpression strain (NB210, *att:mer^+^*) had an overall slower growth rate than the parent strain (Table S1). When switching from acetate to methanol, overexpression of native Mer led to slower growth, lower biomass, and slightly faster flux through methanogenesis (Fig. 5b) as more methyl-H_4_MPT is diverted to methane synthesis.

While flux through methanogenesis enzymes can increase or decrease depending on the carbon substrate, it has also been proposed that methanogenesis is biochemically reversible. The enzymes involved in the Hdr:ACDS:Mer complex are key conserved enzymes thought to be essential for such reversibility to occur (11, 44–46). As the ability to alter the direction of key methanogenesis steps appears essential for *Methanosarcinales* to grow on multiple substrates, the formation of such a complex might be particularly important for metabolic flexibility in *M. acetivorans*. Following affinity purification of NB249, tagged Mer^his-strep^ (36.5 kDa) appears to copurify with untagged Mer (34.7 kDa) (Fig. 1f), indicating the constitutively overexpressed Mer is likely involved in the formation of Mer tetramers, and thus potentially with an Hdr:ACDS:Mer complex.

The HdrED catalytic subunit, HdrD, which was found to be interacting with ACDS in the Hdr:ACDS:Mer complex (19), appears to resemble a fusion protein of the HdrABC catalytic subunit (25, 26). So, while interactions between HdrED and ACDS have been identified, there is also the possibility that HdrABC may bind with ACDS, though evidence of this interaction has not yet been obtained. In previous experiments, overexpression of HdrABC led to an increase in methanogenesis rates under methylotrophic conditions but not acetoclastic and provided further evidence of the importance in regulating gene expression in *M. acetivorans’* ability to respond to changes in substrate availability (31). As each of the reactions catalyzed by the Hdr:ACDS:Mer enzymes is reversible, altering the stoichiometry of this complex by Mer overexpression appears to be important for increasing growth under changing substrate conditions.

When comparing the effects of the tagged overexpression strain (NB249, *att:mer^his-strep+^*) to the untagged version (NB210, *att:mer^+^*), the variations observed could be a result of the His-StrepII tag interfering with the formation of the tetramer complex. Predictive models and protein docking indicate that the tetramer structure of the *att:mer^his-strep+^* strain varies slightly from the native Mer structure (Fig. S2). Although modeling does not predict significant unfolding or other drastic changes, the tag could affect the rate of catalysis and/or formation of protein interactions, such as with Hdr and ACDS. If the tag resulted in inactive protein, we would expect no effect on metabolism or a dominant-negative effect if Mer was involved in substrate channeling with other methanogenesis enzymes. However, we observed that growth is faster on methanol when either the tagged or native untagged versions are overexpressed and no effect when either version of the protein is overexpressed on acetate. This observation suggests both forms of the overexpressed protein are active *in vivo* and that naturally Mer expression is sub-optimal during methylotrophic growth. The effect of the tag is revealed when switching from methanol to acetate or acetate to methanol. Growth rate is slower when overexpressing native Mer (*att:mer^+^*) under all conditions, while overexpression of tagged Mer (*att:mer^his-strep+^*) has no effect from methanol to acetate and a less drastic effect than overexpressing native Mer when switching from acetate to methanol. We interpret these results to suggest that overexpressing native Mer promotes flux through the oxidative branch of the methanogenesis pathway, possibly by forming stable protein complexes that encourage substrate channeling, while the tag may possibly weaken protein interactions between Mer (hetero)oligomers and other proteins, thus resulting in a less drastic phenotype than when native Mer is overexpressed.

Mer overexpression, which diverts CH_3_-H4MPT from the methane pool to instead oxidize F_420_, results in F_420_H_2_ production and increased energy conservation by diverting CH_3_-H_4_MPT and electrons away from acetyl-CoA through Fpo and HdrED. Despite the upregulation of the *Rhodobacter* nitrogen fixation (Rnf) enzyme during acetoclastic growth, ferredoxin oxidation through Rnf and HdrED is still a less efficient way to conserve energy than using F_420_ and F_420_H_2_ oxidoreductase (Fpo). Overall, results reported here are consistent with previous experiments that suggest oxidation of ferredoxin is a rate-limiting step (30) and is poorly coupled to ATP synthesis. When growing on acetate as sole energy source, this effect is enhanced, as the oxidative branch of the C1 pathway is transcribed at a lower rate (46, 47, 48). However, the increase in biomass observed due to Mer overexpression occurs at a slower kinetic rate, presumably to allow the cell to rebalance reducing equivalents and adjust gene expression over several generations.

Overexpression of Mer and the resulting imbalance of substrates and redox carrier pools may also produce transient changes to intracellular cell signaling. *E. coli*, for example, can sense NAD(*P*)H/NAD(*P*) and ATP ratios through the activation of signaling pathways by kinases such as NADK (49, 50) and PFK (51). However, at this time, not enough is known about how *Methanosarcina* cells sense and respond to changes in intracellular metabolite pools to speculate. Future experiments by the research community are encouraged to address these compelling questions.

As Mer is an essential enzyme in the methanogenesis pathway and in the Wood-Ljungdahl (WL) carbon fixation pathway (23, 52, 53), our observations in *M. acetivorans* may have wider implications in methanogens and WL-using microbes in general. As the Hdr:ACDS:Mer complex has been suggested to act as a “router” in sampling methyl-H_4_MPT availability (20), increasing flux through Mer could impact the formation of an ACDS:Mer complex and thus affect conversion of methyl-H_4_MPT to methylene-H_4_MPT. Our observations indicate that control of Mer expression is important for regulating carbon flux through the oxidative branch of methylotrophic and acetoclastic methanogenesis.

## Data Availability

All data used to produce the figures and tables described in the article are provided. Primers, gene parts, and plasmids are provided in Table 1 or in the supplemental material.

## SUPPLEMENTAL MATERIAL

Supplemental material aem.00675-25-s0001.pdf Tables S1 to S5, Fig. S1 to S4, and DNA sequences for pNB746 and pMW1.

ASM does not own the copyrights to Supplemental Material that may be linked to, or accessed through, an article. The authors have granted ASM a non-exclusive, world-wide license to publish the Supplemental Material files. Please contact the corresponding author directly for reuse.
