# Supplementary material for: Mer overexpression in Methanosarcina acetivorans affects growth and methanogenesis during substrate adaptation
Source: Appl Environ Microbiol. 2025 Apr 25;91(5):e00675-25. doi: 10.1128/aem.00675-25 (PMC12093972; doi:10.1128/aem.00675-25)
Supplement: Supplemental material — Tables S1 to S5, Fig. S1 to S4, and DNA sequences for pNB746 and pMW1. [file aem.00675-25-s0001.pdf]

SUPPLEMENTARY INFORMATION

Mer overexpression in *Methanosarcina acetivorans* affects growth and methanogenesis during substrate adaptation

Darla Brennan, Dillon Lieber, Mary Walter, Morgan Price, Nicole R. Buan*
Department of Biochemistry, University of Nebraska-Lincoln

*Please address correspondence to nbuan@unl.edu.

Table S1. Growth data for Mer overexpression mutants.

Substrate	Strain	Doubling time (h)	Std dev	p vs parent
Methanol[a]	parent	9.59	0.608	1
	att:mer^{+}	8.36	0.787	0.002
	att:mer$^{his\text{-}strep+}$	8.85	0.452	0.010
Acetate	parent	47.88	11.82	1
	att:mer^{+}	52.08	15.52	0.589
	att:mer$^{his\text{-}strep+}$	55.84	5.980	0.175
Methanol-to-Acetate[b]	parent	91.19	8.589	1
	att:mer^{+}	125.61	13.22	0.000
	att:mer$^{his\text{-}strep+}$	91.54	16.45	0.958
Acetate-to-Methanol[b]	parent	9.837	1.322	1
	att:mer^{+}	17.26	7.579	0.025
	att:mer$^{his\text{-}strep+}$	12.96	3.899	0.104

Data was obtained from ten biological replicates (n=10).

a. Cultures were adapted to substrate by passaging for 30 generations before measuring growth.
b. Cultures were adapted to original substrate for 30 generations and on a subsequent passage inoculated into media containing the alternate substrate for growth rate measurements.

Table S2. Biomass measurements for Mer overexpression mutants.

Substrate	Strain	Biomass (g)	Std dev	p vs parent
Methanol[a]	parent	0.0126	0.0008	1
	att:mer^{+}	0.0099	0.0015	0.001
	att:mer$^{his\text{-}strep+}$	0.0090	0.0010	0.000
Acetate[a]	parent	0.0059	0.0007	1
	att:mer^{+}	0.0056	0.0007	0.398
	att:mer$^{his\text{-}strep+}$	0.0060	0.0006	0.815
Methanol-to-Acetate[b]	parent	0.0047	0.0010	1
	att:mer^{+}	0.0051	0.0005	0.133
	att:mer$^{his\text{-}strep+}$	0.0053	0.0010	0.042
Acetate-to-Methanol[b]	parent	0.0070	0.0008	1
	att:mer^{+}	0.0059	0.0009	0.022
	att:mer$^{his\text{-}strep+}$	0.0069	0.0007	0.927

Data was obtained from ten biological replicates (n=10).

a. Cultures were adapted to substrate by passaging for 30 generations before measuring biomass (g dry weight per 10 ml culture).
b. Cultures were adapted to original substrate for 30 generations and on a subsequent passage inoculated into media containing the alternate substrate for biomass measurements (g dry weight per 10 ml culture).

Table S3. Methane rates for Mer overexpression mutants.

Substrate	Strain	Methane rate (nmol g^{-1} hr^{-1})	Std dev	p vs parent
Methanol[a]	parent	11.939	2.622	1
	att:mer^{+}	9.339	1.284	0.126
	att:mer$^{his\text{-}strep+}$	10.033	2.856	0.354
Acetate[a]	parent	12.814	2.500	1
	att:mer^{+}	12.290	1.281	0.695
	att:mer$^{his\text{-}strep+}$	16.415	2.005	0.058
Methanol-to-Acetate[b]	parent	5.856	0.912	1
	att:mer^{+}	11.753	1.319	0.000
	att:mer$^{his\text{-}strep+}$	9.792	1.979	0.000
Acetate-to-Methanol[b]	parent	4.004	1.109	1
	att:mer^{+}	5.252	1.128	0.192
	att:mer$^{his\text{-}strep+}$	4.542	1.105	0.460

Data was obtained from two biological and 4-5 technical replicates (n=8-10).

a. Cultures were adapted to substrate by passaging for 30 generations before measuring methane production.
b. Cultures were adapted to original substrate for 30 generations and on a subsequent passage inoculated into media containing the alternate substrate for methane rate measurements.

Table S4. Efficiency and productivity calculations for Mer overexpression mutants.

Metabolic efficiency				
Substrate	Strain	% (k mol^{-1})	Std dev	p vs parent
Methanol[a]	parent	100	17.15	1
	att:mer^{+}	106	18.39	0.672
	att:mer$^{his\text{-}strep+}$	123	28.49	0.284
Acetate[a]	parent	100	16.82	1
	att:mer^{+}	115	14.71	0.210
	att:mer$^{his\text{-}strep+}$	87.2	7.933	0.000
Methanol-to-Acetate[b]	parent	100	16.29	1
	att:mer^{+}	53.6	7.577	0.000
	att:mer$^{his\text{-}strep+}$	61.9	13.65	0.000
Acetate-to-Methanol[b]	parent	100	25.50	1
	att:mer^{+}	138	29.94	0.112
	att:mer$^{his\text{-}strep+}$	121	26.94	0.189
Biomass productivity				
Substrate	Strain	% (g mol^{-1})	Std dev	p vs parent
Methanol[a]	parent	100	20.35	1
	att:mer^{+}	97.1	13.05	0.820
	att:mer$^{his\text{-}strep+}$	88.2	26.13	0.499
Acetate[a]	parent	100	16.83	1
	att:mer^{+}	91.9	9.333	0.433
	att:mer$^{his\text{-}strep+}$	73.1	8.961	0.032
Methanol-to-Acetate[b]	parent	100	18.26	1
	att:mer^{+}	52.5	5.931	0.005
	att:mer$^{his\text{-}strep+}$	75.7	10.78	0.058
Acetate-to-Methanol[b]	parent	100	22.56	1
	att:mer^{+}	63.7	13.72	0.035
	att:mer$^{his\text{-}strep+}$	79.8	19.72	0.215

Values were calculated from data shown in Tables S1-S3, respectively.

a. Cultures were adapted to substrate by passaging for 30 generations before measuring methane production.
b. Cultures were adapted to original substrate for 30 generations and on a subsequent passage inoculated into media containing the alternate substrate for methane rate measurements.

Table S5. Cell area of Mer overexpression strains compared to parent strain cells.

Substrate		***Cell area (um²)***	***Std dev***	***p vs. parent***
Methanol[a]	parent	3.522	0.567	1
	att:mer^{+}	2.673	0.484	6.92E-40
	att:mer$^{+his\text{-}strep}$	3.128	0.457	4.01E-16
Methanol-to-Acetate[b]	parent	3.394	1.120	1
	att:mer^{+}	2.174	0.573	1.73E-15
	att:mer$^{+his\text{-}strep}$	2.815	0.764	0.000214
Acetate[a]	parent	2.615	0.887	1
	att:mer^{+}	3.090	0.494	0.000423
	att:mer$^{+his\text{-}strep}$	2.067	0.714	6.67E-06
Acetate-to-Methanol[b]	parent	3.172	0.708	1
	att:mer^{+}	2.693	0.587	4.79E-10
	att:mer$^{+his\text{-}strep}$	2.513	0.468	1.42E-31

Cell sizes were measured from triplicate biological replicates: MeOH (n>100 cells per strain), MtoA (n>70 cells per strain), Acetate (n>40 cells per strain), AtoM (n>100 cells per strain).

a. Cultures were adapted to substrate by passaging for 30 generations.
b. Cultures were adapted to original substrate for 30 generations and on a subsequent passage inoculated into media containing the alternate substrate.

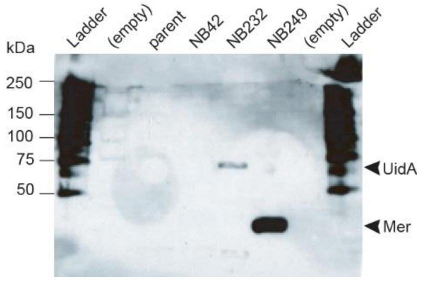

Figure S1. Detection of Mer overexpression. Immunoblot incubated with Strep-II antibody for verification of tagged Mer (*att:mer*$^{his\text{-}strep+}$). Crude cell lysate from parent (NB34), *att:uidA*$^{his\text{-}strep}$ (NB232), and *att:mer*$^{+his\text{-}strep}$ (NB249) was separated by 4% stacking/12.5% separating SDS-PAGE. NB232 is a positive control strain expressing tagged UidA enzyme.

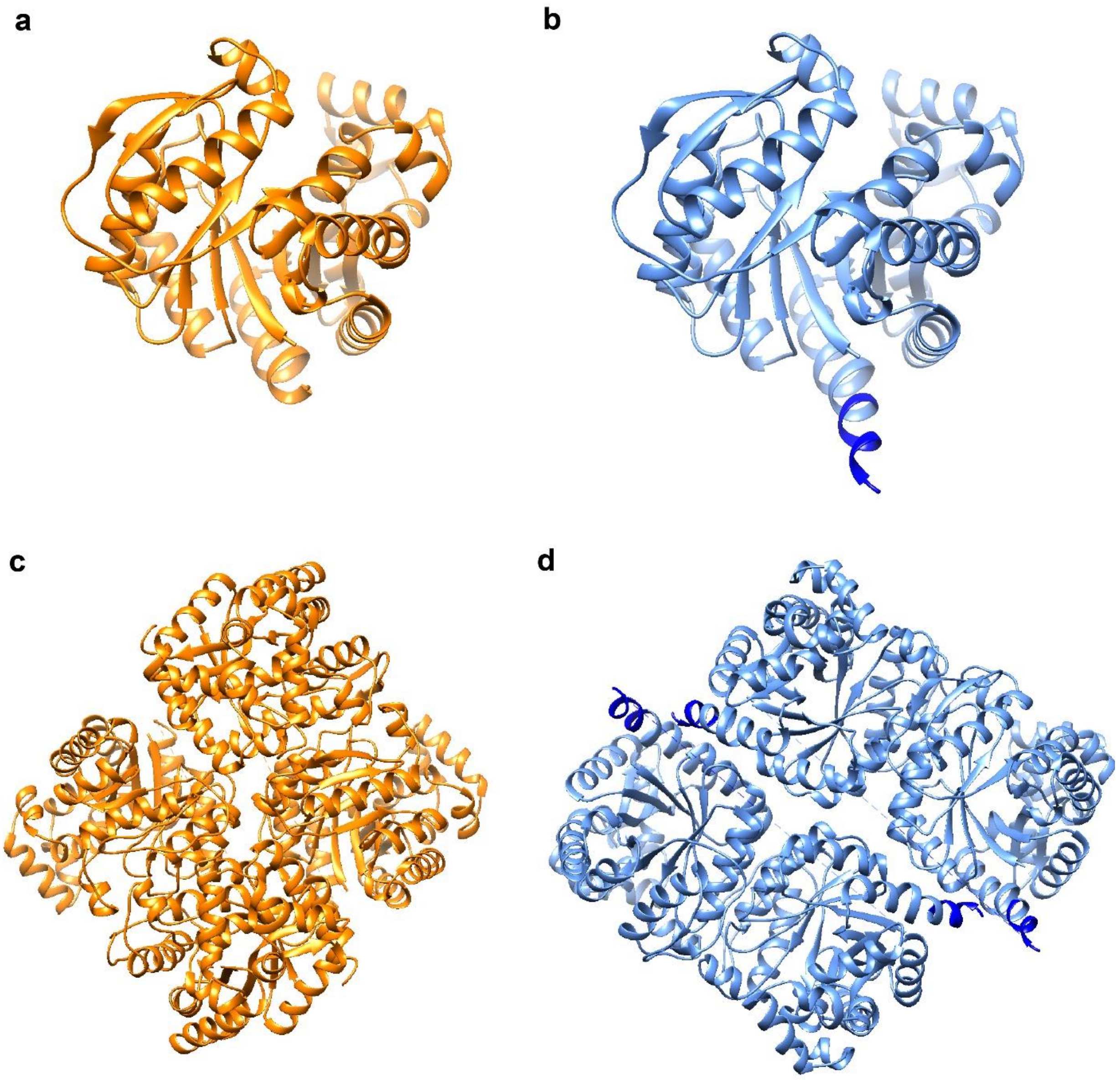

Figure S2. Structural models of native Mer and tagged Mer$^{\text{his-strep}}$ protein. Panel a, Phyre2 homology model of Mer, MA3733. Panel b, Phyre2 homology model of Mer$^{\text{his-strep}}$ highlighted in dark blue. Panel c, Predicted native Mer tetramer in *M. acetivorans* docked using SymmDock server (https://bioinfo3d.cs.tau.ac.il/SymmDock) with a symmetry of 4 indicated. Panel d, Predicted Mer$^{\text{his-strep}}$ tetramer in *M. acetivorans* docked using SymmDock server with a symmetry of 4 indicated.

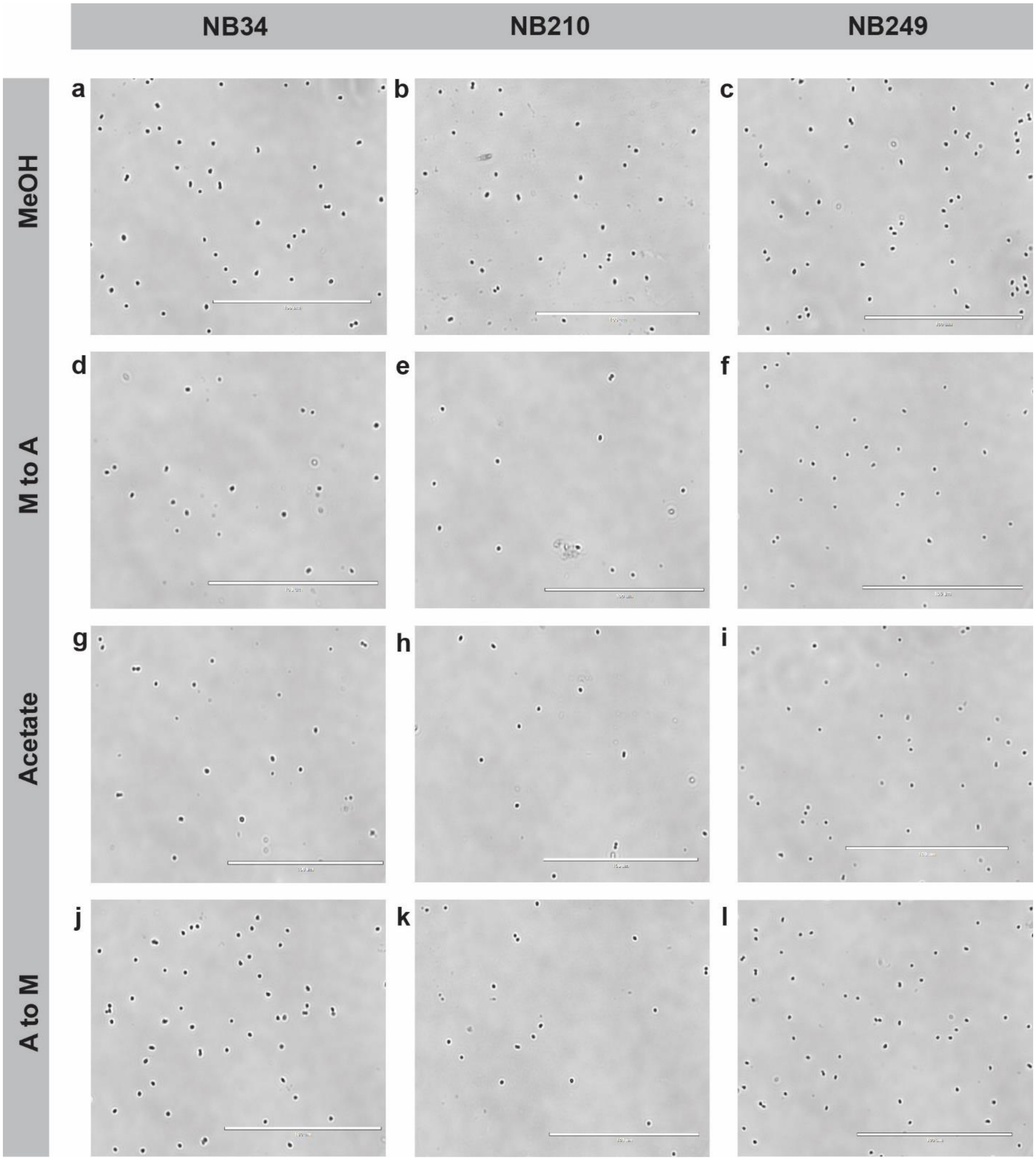

Figure S3. Microscopy of parent and Mer overexpression strain cells. Parent (NB34), *att:mer*$^{+}$ (N210), and *att:mer*$^{+his\text{-}strep}$ (N249) cells were grown in each carbon source condition to early stationary phase (OD_{600} ~0.7 methanol grown or OD_{600} ~0.11 for acetate grown cells) before visualizing 10μL cell culture using an EVOS FL Digital Microscope (Thermo Fisher Scientific, Waltham, MA). Panel a-c, Methanol grown NB34, NB210, and NB249 respectively. Panel d-f, Methanol adapted and switched to acetate NB34, N210, and NB249 grown cells respectively. Panel g-i, Acetate grown NB34, NB210, NB249 cells respectively. Panel j-l,

Acetate adapted and switched to methanol grown NB34, NB210, and NB249 cells. The scale bar represents 100µm.

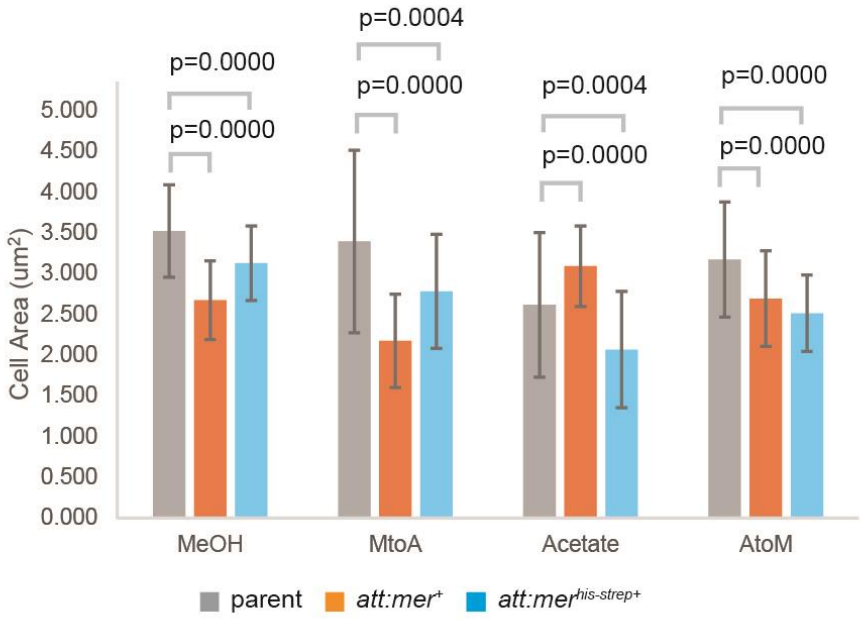

Figure S4. Cell sizes of Mer overexpression strains compared to parent strain. Cell area (µm^2) of parent and overexpression strains when adapted to methanol or acetate and when switched from methanol to acetate (MtoA) or acetate to methanol (AtoM). Cell sizes were measured from triplicate biological replicates: MeOH (n>100 cells per strain), MtoA (n>70 cells per strain), Acetate (n>40 cells per strain), AtoM (n>100 cells per strain).

DNA Sequence for pNB746

tctagttgtctgctcccggcatccgcttacagacaagctgtgaccgtctccgggagctgcatgtgtcagaggttttcaccgtcatcaccgaaa
cgcgcgagacgaaagggcctcgtgatacgcctatttttataggttaatgtcatgataataatggtttcttagacgtcaggtggcacttttcgggg
aaatgtgcgcggaacccctatttgtttatttttctaaatacattcaaatatgtatccgctcatgagacaataaccctgataaatgcttcaataatatt
gaaaaaggaagagtatgagtattcaacatttccgtgtcgcccttattcccttttttgcggcattttgccttcctgtttttgctcacccagaaacgctg
gtgaaagtaaaagatgctgaagatcagttgggtgcacgagtgggttacatcgaactggatctcaacagcggtaagatccttgagagttttcg
ccccgaagaacgttttccaatgatgagcacttttaaagttctgctatgtggcgcggtattatcccgtattgacgccgggcaagagcaactcggt
cgccgcatacactattctcagaatgacttggttgagtactcaccagtcacagaaaagcatcttacggatggcatgacagtaagagaattatgc
agtgctgccataaccatgagtgataacactgcggccaacttacttctgacaacgatcggaggaccgaaggagctaaccgcttttttgcacaa
catgggggatcatgtaactcgccttgatcgttgggaaccggagctgaatgaagccataccaaacgacgagcgtgacaccacgatgcctgt
agcaatggcaacaacgttgcgcaaactattaactggcgaactacttactctagcttcccggcaacaattaatagactggatggaggcggata
aagttgcaggaccacttctgcgctcggcccttccggctggctggtttattgctgataaatctggagccggtgagcgtgggtctcgcggtatca
ttgcagcactggggccagatggtaagccctcccgtatcgtagttatctacacgacggggagtcaggcaactatggatgaacgaaatagaca
gatcgctgagataggtgcctcactgattaagcattggtaactgtcagaccaagtttactcatatatactttagattgatttaaaacttcatttttaattt
aaaaggatctaggtgaagatcctttttgataatctcatgaccaaaatcccttaacgtgagttttcgttccactgagcgtcagaccccgtagaaaa
gatcaaaggatcttcttgagatcctttttttctgcgcgtaatctgctgcttgcaaacaaaaaaaccaccgctaccagcggtggtttgtttgccgga
tcaagagctaccaactctttttccgaaggtaactggcttcagcagagcgcagataccaaatactgttcttctagtgtagccgtagttaggccac
cacttcaagaactctgtagcaccgcctacatacctcgctctgctaatcctgttaccagtggctgctgccagtggcgataagtcgtgtcttaccg
ggttggactcaagacgatagttaccggataaggcgcagcggtcgggctgaacggggggttcgtgcacacagcccagcttggagcgaac
gacctacaccgaactgagatacctacagcgtgagctatgagaaagcgccacgcttcccgaagggagaaaggcggacaggtatccggtaa
gcggcagggtcggaacaggagagcgcacgagggagcttccagggggaaacgcctggtatctttatagtcctgtcgggtttcgccacctct
gacttgagcgtcgatttttgtgatgctcgtcaggggggcggagcctatggaaaaacgccagcaacgcggcctttttacggttcctggcctttt
gctggccttttgctcacatgttctttcctgcgttatcccctgattctgtggataaccgtattaccgcctttgagtgagctgataccgctcgccgcag
ccgaacgaccgagcgcagcgagtcagtgagcgaggaagcggaagagcgcccaatacgcaaaccgcctctccccgcgcgttggccgat
tcattaatgcagctggcacgacaggtttcccgactggaaagcgggcagtgagcgcaacgcaattaatgtgagttagctcactcattaggcac
cccaggctttacactttatgcttccggctcgtatgttgtgtggaattgtgagcggataacaatttcacacaggaaacagctatgaccatgattac
gccaagcttgcatgctcatgctccaggtttcctgtccttcgggcacctcgacgtcggcggtgacggtgaagccgagccgctcgtagaaggg
gaggttgcggggcgcggaggtctccaggaaggcgggcaccccggcgcgctcggccgcctccactccggggagcacgacggcgctgc
ccagacccttgccctggtggtcgggcgagacgccgacggtggccaggaaccacgcgggctccttgggccggtgcggcgccaggaggc
cttccatctgttgctgcgcggccagccgggaaccgctcaactcggccatgcgcgggccgatctcggcgaacaccgcccccgcttcgacg
ctctccggcgtggtccagaccgccaccgcggcgccgtcgtccgcgacccacaccttgccgatgtcgagcccgacgcgcgtgaggaaga
gttcttgcagctcggtgacccgctcgatgtggcggtccgggtcgacggtgtggcgcgtggcggggtagtcggcgaacgcggcggcgag
ggtgcgtacggcccgggggacgtcgtcgcgggtggcgaggcgcaccgtgggtttatattcggtcatgagaatcactctagttcctattttttt
gatatatacatcataacattactctatgtatatatattcactttttcattaacattaaatagaaaagtttatatataagatgttaataacacaataatttg
aatttgaatactcaaaaaatgggctttaatatataaaattaagatgaaaatagatgatttttttaaaaaaatgttattattatatctcaatatctaaatatt
agattaatattaattattacccaaatatttcaatgaatatttagttttgaatagtatattacgaatagggcgtttttttattacctactactattttccgaag
attttttaagactctcttaaaattaatcatcctctagaggcgcgccaatacgcaaaccgactagacttaattaagatccggcgcgcccccgggt
accgagctcgaattcactggccgtcgttttacaacgtcgtgactgggaaaaccctggcgttacccaacttaatcgccttgcagcacatccccc
tttcgccagctggcgtaatagcgaagaggcccgcaccgatcgcccttcccaacagttgcgcagcctgaatggcgaatggcgcctgatgcg
gtattttctccttacgcatctgtgcggtatttcacacctcatttatcggagaacacaaaagatttaagtaccttctaaacgaatgagatttcattggg
aatagtggacactcgagtaggtgaccagtcccaaaatgattttaataaattaaggaggaaattcatatgtgaagttcggaatcgaatttgtgcc
gagcgatcctgctttaaagatcgcatattacgcaaagctctcagaacagcagggattcgactacgtctggatcactgatcactacaacaacc
gtgacgtatattccactcttaccgttctcgctctgaataccaacagcatcaagattggctccggtgtcacaaactcctataccaggaaccctgc
aattacagcatcaagcattgcttccatcgccgaaatttccggcggcagggcagtcctcggactcggacccggagacaaggcaaccttcgat
gccatgggcattgcctgggaaaagcctcttgcaaccaccaaagaagcaatccaggcaatcagagatttcattgccggcaaaaaagtctcca
tggacggcgaaatggtcaagttcgcaggtgcaaagctcgcttttaaagctggaaatgttccaatttacatgggtgctcagggtcccaagatgc

tcgaacttgccggtgaagttgcagacggcgtcctgatcaacgcttcccatccgaaggactttgaagtcgccgtggaacagatccgcaaggg
cgctgaaaaagtcggtcgcgacccaagcgaagtcgatgtcaccgcatacgcttgcttctccattgacaaagaccccgcaaaagccatcaac
gctgcaaaagtagtggtcgccttcatcgttgcaggttcccctgaccttgtccttgaaaggcacggaatccctgtcgacgccaagaagcagat
cggtgacgctattgccaaaggagatttcggagccctcatgggtggacttgttacccccagatgatcgaagcctttgcaatctgcggaactc
ctgaggactgcatgaagaggattaaagatcttgaggcaatcggagtcacccagattgtcgccggatccccgatcggtcctgagaaagaaa
aagcaataaagcttataggcaaagagatcattgcaaagatgttggatccaagcttgggccctcgcgactcgagacaccaccatcatcatcac
caccattggtcccatccccaatttgaaaagtagttaatctagttgacgcgccctgacgggactagaatgaatcaacaactctcctggcgcacc
atcgtcggctacagcctcggtgacgtcgccaataacttcgccttcgcaatgggggcgctcttcctgttgagttactacaccgacgtcgctggc
gtcggtgccgctgcggcgggcaccatgctgttactggtgcgggtattcgatgccttcgccgacgtctttgccggacgagtggtggacagtgt
gaatatccgctggggaaaattccgcccgtttttactcttcggtactgcgccgttaatgatcagatccgagctcaagcttcttgataacttcgtata
atgtatgctatacgaagttatccctttagtgagggttaattaagcggccgcccgggccggccatttaaatgcatgcgacttccgaaaaaacag
caaagaaaagccagtatggaaaaaatagacaaaaagtaggctaaaaggcctactctgttttaaactgttgaatttattgagttcgagtgaggtg
gagtacgcgcccggggagcccaagggcacgccctggcacccgcaccgcggatcgatcgaattctcgaccaattctcatgtttgacagctt
atcatcgaatttctgccattcatccgcttattatcacttattcaggcgtagcaaccaggcgtttaagggcaccaataactgccttaaaaaaattac
gccccgccctgccactcatcgcagtactgttgtaattcattaagcattctgccgacatggaagccatcacaaacggcatgatgaacctgaatc
gccagcg

DNA Sequence pMW1

tctagttgtctgctcccggcatccgcttacagacaagctgtgaccgtctccgggagctgcatgtgtcagaggttttcaccgtcatcaccgaaa
cgcgcgagacgaaagggcctcgtgatacgcctatttttataggttaatgtcatgataataatggtttcttagacgtcaggtggcacttttcgggg
aaatgtgcgcggaacccctatttgtttatttttctaaatacattcaaatatgtatccgctcatgagacaataaccctgataaatgcttcaataatatt
gaaaaaggaagagtatgagtattcaacatttccgtgtcgcccttattcccttttttgcggcattttgccttcctgtttttgctcacccagaaacgctg
gtgaaagtaaaagatgctgaagatcagttgggtgcacgagtgggttacatcgaactggatctcaacagcggtaagatccttgagagttttcg
ccccgaagaacgttttccaatgatgagcacttttaaagttctgctatgtggcgcggtattatcccgtattgacgccgggcaagagcaactcggt
cgccgcatacactattctcagaatgacttggttgagtactcaccagtcacagaaaagcatcttacggatggcatgacagtaagagaattatgc
agtgctgccataaccatgagtgataacactgcggccaacttacttctgacaacgatcggaggaccgaaggagctaaccgcttttttgcacaa
catgggggatcatgtaactcgccttgatcgttgggaaccggagctgaatgaagccataccaaacgacgagcgtgacaccacgatgcctgt
agcaatggcaacaacgttgcgcaaactattaactggcgaactacttactctagcttcccggcaacaattaatagactggatggaggcggata
aagttgcaggaccacttctgcgctcggcccttccggctggctggtttattgctgataaatctggagccggtgagcgtgggtctcgcggtatca
ttgcagcactggggccagatggtaagccctcccgtatcgtagttatctacacgacggggagtcaggcaactatggatgaacgaaatagaca
gatcgctgagataggtgcctcactgattaagcattggtaactgtcagaccaagtttactcatatatactttagattgatttaaaacttcatttttaattt
aaaaggatctaggtgaagatcctttttgataatctcatgaccaaaatcccttaacgtgagttttcgttccactgagcgtcagaccccgtagaaaa
gatcaaaggatcttcttgagatcctttttttctgcgcgtaatctgctgcttgcaaacaaaaaaaccaccgctaccagcggtggtttgtttgccgga
tcaagagctaccaactctttttccgaaggtaactggcttcagcagagcgcagataccaaatactgttcttctagtgtagccgtagttaggccac
cacttcaagaactctgtagcaccgcctacatacctcgctctgctaatcctgttaccagtggctgctgccagtggcgataagtcgtgtcttaccg
ggttggactcaagacgatagttaccggataaggcgcagcggtcgggctgaacggggggttcgtgcacacagcccagcttggagcgaac
gacctacaccgaactgagatacctacagcgtgagctatgagaaagcgccacgcttcccgaagggagaaaggcggacaggtatccggtaa
gcggcagggtcggaacaggagagcgcacgagggagcttccagggggaaacgcctggtatctttatagtcctgtcgggtttcgccacctct
gacttgagcgtcgatttttgtgatgctcgtcaggggggcggagcctatggaaaaacgccagcaacgcggcctttttacggttcctggcctttt
gctggccttttgctcacatgttctttcctgcgttatcccctgattctgtggataaccgtattaccgcctttgagtgagctgataccgctcgccgcag
ccgaacgaccgagcgcagcgagtcagtgagcgaggaagcggaagagcgcccaatacgcaaaccgcctctccccgcgcgttggccgat
tcattaatgcagctggcacgacaggtttcccgactggaaagcgggcagtgagcgcaacgcaattaatgtgagttagctcactcattaggcac
cccaggctttacactttatgcttccggctcgtatgttgtgtggaattgtgagcggataacaatttcacacaggaaacagctatgaccatgattac
gccaagcttgcatgctcatgctccaggtttcctgtccttcgggcacctcgacgtcggcggtgacggtgaagccgagccgctcgtagaaggg
gaggttgcggggcgcggaggtctccaggaaggcgggcaccccggcgcgctcggccgcctccactccggggagcacgacggcgctgc
ccagacccttgccctggtggtcgggcgagacgccgacggtggccaggaaccacgcgggctccttgggccggtgcggcgccaggaggc
cttccatctgttgctgcgcggccagccgggaaccgctcaactcggccatgcgcgggccgatctcggcgaacaccgcccccgcttcgacg
ctctccggcgtggtccagaccgccaccgcggcgccgtcgtccgcgacccacaccttgccgatgtcgagcccgacgcgcgtgaggaaga
gttcttgcagctcggtgacccgctcgatgtggcggtccgggtcgacggtgtggcgcgtggcggggtagtcggcgaacgcggcggcgag
ggtgcgtacggcccgggggacgtcgtcgcgggtggcgaggcgcaccgtgggtttatattcggtcatgagaatcactctagttcctattttttt
gatatatacatcataacattactctatgtatatatattcactttttcattaacattaaatagaaaagtttatatataagatgttaataacacaataatttg
aatttgaatactcaaaaaatgggctttaatatataaaattaagatgaaaatagatgatttttaaaaaaatgttattattatatctcaatatctaaatatt
agattaatattaattattacccaaatatttcaatgaatatttagttttgaatagtatattacgaatagggcgtttttttattacctactactattttccgaag
atttttaagactctcttaaaattaatcatcctctagaggcgcgccaatacgcaaaccgactagacttaattaagatccggcgcgcccccgggt
accgagctcgaattcactggccgtcgttttacaacgtcgtgactgggaaaaccctggcgttacccaacttaatcgccttgcagcacatccccc
tttcgccagctggcgtaatagcgaagaggcccgcaccgatcgcccttcccaacagttgcgcagcctgaatggcgaatggcgcctgatgcg
gtattttctccttacgcatctgtgcggtatttcacacctcatttatcggagaacacaaaagatttaagtaccttctaaacgaatgagatttcattggg
aatagtggacactcgagtaggtgaccagtcccaaaatgattttaataaattaaggaggaaattcatatgtgaagttcggaatcgaatttgtgcc
gagcgatcctgctttaaagatcgcatattacgcaaagctctcagaacagcagggattcgactacgtctggatcactgatcactacaacaacc
gtgacgtatattccactcttaccgttctcgctctgaataccaacagcatcaagattggctccggtgtcacaaactcctataccaggaaccctgc
aattacagcatcaagcattgcttccatcgccgaaatttccggcggcagggcagtcctcggactcggacccggagacaaggcaaccttcgat
gccatgggcattgcctgggaaaagcctcttgcaaccaccaaagaagcaatccaggcaatcagagatttcattgccggcaaaaaagtctcca
tggacggcgaaatggtcaagttcgcaggtgcaaagctcgcttttaaagctggaaatgttccaatttacatgggtgctcagggtcccaagatgc

tcgaacttgccggtgaagttgcagacggcgtcctgatcaacgcttcccatccgaaggactttgaagtcgccgtggaacagatccgcaaggg
cgctgaaaaagtcggtcgcgacccaagcgaagtcgatgtcaccgcatacgcttgcttctccattgacaaagaccccgcaaaagccatcaac
gctgcaaaagtagtggtcgccttcatcgttgcaggttcccctgaccttgtccttgaaaggcacggaatccctgtcgacgccaagaagcagat
cggtgacgctattgccaaaggagatttcggagccctcatgggtggacttgttaccccccagatgatcgaagcctttgcaatctgcggaactc
ctgaggactgcatgaagaggattaaagatcttgaggcaatcggagtcacccagattgtcgccggatccccgatcggtcctgagaaagaaa
aagcaataaagcttataggcaaagagatcattgcaaagatgttggatccaagcttgggccctcgcgactcgagacaccaccatcatcatcac
caccattggtcccatccccaatttgaaaagtagggatccaagcttgggccctcgcgactcgagacaccaccatcatcatcaccaccattggt
cccatccccaatttgaaaagtagttaatctagttgacgcgccctgacgggactagaatgaatcaacaactctcctggcgcaccatcgtcggct
acagcctcggtgacgtcgccaataacttcgccttcgcaatgggggcgctcttcctgttgagttactacaccgacgtcgctggcgtcggtgcc
gctgcggcgggcaccatgctgttactggtgcgggtattcgatgccttcgccgacgtctttgccggacgagtggtggacagtgtgaatatccg
ctggggaaaattccgcccgtttttactcttcggtactgcgccgttaatgatcagatccgagctcaagcttcttgataacttcgtataatgtatgcta
tacgaagttatccctttagtgagggttaattaagcggccgcccgggccggccatttaaatgcatgcgacttccgaaaaaacagcaaagaaaa
gccagtatggaaaaaatagacaaaaagtaggctaaaaggcctactctgttttaaactgttgaatttattgagttcgagtgaggtggagtacgcg
cccggggagcccaagggcacgccctggcacccgcaccgcggatcgatcgaattctcgaccaattctcatgtttgacagcttatcatcgaatt
tctgccattcatccgcttattatcacttattcaggcgtagcaaccaggcgtttaagggcaccaataactgccttaaaaaaattacgccccgccct
gccactcatcgcagtactgttgtaattcattaagcattctgccgacatggaagccatcacaaacggcatgatgaacctgaatcgccagcg
